# Purple Sweet Potato Leaf Extract Induces Apoptosis and Reduces Inflammatory Adipokine Expression in 3T3-L1 Differentiated Adipocytes

**DOI:** 10.1155/2015/126302

**Published:** 2015-06-11

**Authors:** Shou-Lun Lee, Ting-Yu Chin, Ssu-Chieh Tu, Yu-Jie Wang, Ya-Ting Hsu, Ming-Ching Kao, Yang-Chang Wu

**Affiliations:** ^1^Department of Biological Science and Technology, College of Life Sciences, China Medical University, 91 Hsueh-Shih Road, Taichung 40402, Taiwan; ^2^Department of Bioscience Technology, Chung Yuan Christian University, 200 Chung Pei Road, Zhongli District, Taoyuan City 32023, Taiwan; ^3^School of Pharmacy, College of Pharmacy, China Medical University, 91 Hsueh-Shih Road, Taichung 40402, Taiwan; ^4^Chinese Medicine Research and Development Center, China Medical University Hospital, 2 Yude Road, Taichung 40447, Taiwan; ^5^Center for Molecular Medicine, China Medical University Hospital, 2 Yude Road, Taichung 40447, Taiwan; ^6^Graduate Institute of Natural Products, Kaohsiung Medical University, 100 Shih Chuan 1st Road, Kaohsiung 80708, Taiwan

## Abstract

*Background*. Purple sweet potato leaves (PSPL) are widely grown and are considered a healthy vegetable in Taiwan. PSPL contain a high content of flavonoids, and the boiling water-extracted PSPL (PSPLE) is believed to prevent metabolic syndrome. However, its efficacy has not yet been verified. Therefore, we investigated the effect of PSPLE on adipocytes. *Methods*. The differentiated 3T3-L1 cells used in this study were derived from preadipocytes that were differentiated into adipocytes using an adipogenic agent (insulin, dexamethasone, and 3-isobutyl-1-methylxanthine); approximately 90% of the cells were differentiated using this method. *Results*. Treating the differentiated 3T3-L1 cells with PSPLE caused a dose-dependent decrease in the number of adipocytes rather than preadipocytes. In addition, treatment with PSPLE resulted in apoptosis of the differentiated 3T3-L1 cells as determined by DAPI analysis and flow cytometry. PSPLE also increased the expression of cleaved caspase-3 and poly ADP-ribose polymerase (PARP). Furthermore, PSPLE induced downregulation of interleukin-6 (IL-6) and tumor necrosis factor-*α* (TNF-*α*) gene expression in the differentiated 3T3-L1 cells. *Conclusions*. These results suggest that PSPLE not only induced apoptosis but also downregulated inflammation-associated genes in the differentiated 3T3-L1 cells.

## 1. Introduction

A report from the World Health Organization (WHO) indicates that at least 2.8 million people die each year from the consequences of being overweight or obese; excess weight leads to adverse metabolic effects on blood pressure, cholesterol, triglycerides, and insulin resistance [1]. The treatment and prevention of overweight and obesity are one of the most important public health problems.

Adipose tissue is a specialized type of loose connective tissue which contains adipocytes, stromal vascular fraction, and immune cells. Overweight results from adipocyte hypertrophy (cell size increase) and hyperplasia (cell number increase) show that both contribute to adipose tissue expansion [2, 3]. Adipose tissue is an accepted endocrine organ, secreting various adipokines that are involved in the regulation of insulin resistance and metabolic syndrome [4]. Adipokines include classic proinflammatory proteins such as tumor necrosis factor-*α* (TNF-*α*) and interleukin-6 (IL-6), both secreted by adipocytes but synthesized also by immune cells infiltrating white adipose tissue (WAT) such as macrophages [4]. Reports IL-6 and TNF-*α* are the major inflammatory adipokines that impair insulin signaling and its actions that result in the associated development of insulin resistance and type 2 diabetes [5–7]. Sorisky et al. have shown that apoptosis of preadipocytes and adipocytes is a potent regulatory mechanism for adipose tissue mass [8]. Apoptosis is programmed cell death. Reports indicate that there are two main apoptotic pathways, the extrinsic and intrinsic pathways, as well as a perforin/granzyme pathway [9]. These main apoptotic pathways converge to activate caspase-3 and result in several biochemical modifications, including protein cleavage, chromatin condensation, DNA fragmentation, and the formation of apoptotic bodies [10].

3T3-L1 cells are widely and frequently used adipocytes* in vitro*. The key feature of mature adipocytes is the accumulation of oil droplets in the cytoplasm [2]. Therefore, we investigated the effect of the purple sweet potato leaf extract (PSPLE) on differentiated 3T3-L1 cells.

The purple sweet potato (PSP) is planted extensively in Taiwan. Ju et al. have shown that PSP extract has antilipogenic, anti-inflammatory, and lipolytic effects on differentiated 3T3-L1 cells and has radical scavenging and reducing activity [11]. The hot water extract of purple sweet potato leaves (PSPL) has been used to prevent obesity and metabolic syndrome in traditional folk medicine. Reports indicate that PSPL are rich in flavonoids [12], which have been shown to suppress antioxidant [13], inflammatory [14], and carcinogenic effects [15]. In addition, clinical research has shown that the consumption of PSPL can modulate antioxidative status and immune responses [16, 17]. However, it has not been proven that PSPL can ameliorate metabolic syndrome. Our previous studies have shown that the hot water extract of PSPL inhibits cell proliferation of 3T3-L1 preadipocytes and inhibits adipogenesis at a later stage of the differentiation [18]. This study was designed to investigate the effects of PSPLE on mature 3T3-L1 adipocytes. We found that the extract was able to induce apoptosis of adipocytes and reduce the expression of the inflammation-associated genes IL-6 and TNF-*α*. Therefore, we speculate that PSPLE could prevent obesity and metabolic syndrome.

## 2. Methods

### 2.1. Chemicals and Reagents

3T3-L1 cells (ATCC Cat. number CL-173) were purchased from the Bioresource Collection and Research Centre (Food Industry Research and Development Institute, Hsinchu, Taiwan). Anti-*β*-actin antibodies, 4,6-diamidino-2-phenylindole dihydrochloride (DAPI), dexamethasone (DEX), formaldehyde, insulin, 3-isobutyl-1-methylxanthine (IBMX), Oil Red O, propidium iodide (PI), and Triton X-100 were purchased from Sigma-Aldrich (Missouri, USA). Anti-caspase-3 and anti-PARP antibodies were purchased from Cell Signaling Technology (MA, USA). Fetal bovine serum (FBS) was purchased from Thermo Fisher Scientific Inc. (NY, USA). The PCR Amplification Kit was purchased from the Takara Bio Inc. (Shiga, Japan). Percoll was purchased from GE Healthcare Bio-Sciences AB (Uppsala, Sweden). Trypan blue solution (0.5%) was purchased from BioWest (Nuaille, France). All other reagents were purchased from Invitrogen (CA, USA).

### 2.2. Preparation of PSPLE

The purple sweet potato leaves (PSPL,* Ipomoea batatas* Lam.) used in this study were recognized and authenticated by the National Plant Genetic Resources Center of Taiwan Agricultural Research Institute with the account number Pin 375. The freshly cleaned leaves (100 grams) were added to 1 L of boiling H_2_O for 1 h. Then, the PSPL extract (PSPLE) was filtered, lyophilized, and stored at −20°C. The hot water extracts of 100 g freshly cleaned leaves yield 4 g powdered PSPLE. The powdered PSPLE was dissolved in water, and a stock solution was freshly prepared at concentration of 1 g/mL before cell culture experiments.

### 2.3. Cell Culture

3T3-L1 cells were cultured from preadipocytes and differentiated into adipocytes as previously described, with minor modifications [19]. Briefly, preadipocytes were grown in Dulbecco's modified Eagle's medium (DMEM) with high glucose containing 10% (v/v) FBS, 100 U/mL penicillin, and 100 *μ*g/mL streptomycin in plates (10^5^ cells/mL) at 37°C in a humidified atmosphere of 10% CO_2_. The medium was changed every 2 days. Then, 2 days after confluence (designated day 0), the medium was replaced with DMEM containing adipogenic agents (1.7 *μ*M insulin, 0.25 *μ*M DMX, and 0.5 mM IBMX) for 3 days. Cells were then grown in DMEM containing 10% FBS, and the medium was changed every 2 days until day 9. Differentiated cells were used for experimentation on day 9 in which cells were purified by Percoll density gradient centrifugation (see below). The differentiated 3T3-L1 cells were cultured on collagen-coated dishes overnight and then were treated with different concentrations of PSPLE for 72 h.

### 2.4. Separation of Adipocytes and Preadipocytes

The method of gradient centrifugation was performed in accordance with a previously described procedure, with minor modifications [20]. Briefly, the cell suspension was loaded on top of a 25/50% Percoll gradient at a proportion of 1 : 2 : 2 and mixing was avoided. After centrifugation at 1,800 ×g for 15 min at 4°C, the suspension was divided into 3 layers. The middle layer and the lower layer represented differentiated and undifferentiated cells, respectively. These cells were stained with Trypan blue or Oil Red O [21].

### 2.5. DAPI Staining

The differentiated 3T3-L1 cells were cultured in 6-well plates (2 × 10^5^ cells/well) and treated with or without PSPLE for 72 h. Cells were washed with PBS, fixed with 4% formaldehyde for 10 min, and washed repeatedly with PBS. Cells in each well were stained with DAPI for 15 min before fixation with 0.1% Triton X-100 for 15 min. The chromatin changes were examined by fluorescence microscopy.

### 2.6. Apoptosis Analysis by Flow Cytometry

The differentiated 3T3-L1 cells were cultured in 6-well plates (2 × 10^5^ cells/well) and treated with or without PSPLE for 72 h. Cells were collected by trypsinization, washed twice with ice-cold PBS, and then fixed in ice-cold 75% ethanol overnight at −20°C. Next, cells were washed with PBS and resuspended in 1 mL of DNA staining solution (20 *μ*g/mL propidium iodide (PI), 0.1% Triton-X 100, and 100 *μ*g/mL ribonuclease A (RNase A) in PBS) for 30 min in the dark. Then, cells were analyzed by flow cytometry, and the cell cycle was analyzed using the ModTid LT3.0 software program. In addition, apoptosis was also determined using annexin-V/PI double staining with an annexin-V-FITC apoptosis detection kit (BD Biosciences Pharmingen, CA, USA). Cells were analyzed using the Becton-Dickinson FACSCanto and BD CellQuest Pro software programs.

### 2.7. Western Blot Analysis

The differentiated 3T3-L1 cells were treated as indicated, detached and thoroughly washed with PBS, and then lysed in ice-cold lysis buffer. Following centrifugation at 13,000 ×g for 10 min at 4°C, the supernatants (50 *μ*g protein) were boiled with reducing sample buffer for 5 min, subjected to electrophoresis, and then transferred onto a PVDF membrane. The membrane was blocked with 1% BSA in PBS containing 0.1% Tween-20 (PBST) for 1 h at room temperature and then washed with PBST. Next, the membrane was incubated with a primary antibody; finally, the membrane was incubated with a secondary antibody conjugated to horseradish peroxidase (HRP) for 1 h. An enhanced chemiluminescence (ECL) kit (Amersham Biosciences, IL, or Millipore, MA) was used for detection. The relative intensity of the immunoreactive bands was assessed using ImageJ software.

### 2.8. Reverse Transcription-PCR (RT-PCR)

The differentiated 3T3-L1 cells were plated in 35 mm dishes (2 × 10^5^ cells/dish) and treated with or without PSPLE for 72 h. Then, cells were detached and thoroughly washed with cold PBS. RNA was extracted using TRIzol Reagent according to the manufacturer's protocol (Invitrogen, CA, USA). Total RNA (1 *μ*g) was reverse-transcribed using a SuperScript II Reverse Transcriptase Kit (Invitrogen, CA, USA). Aliquots of cDNA were subjected to polymerase chain reaction (PCR) with a PCR Amplification Kit (Takara Bio Inc., Shiga, Japan). Conditions for PCR included initial denaturation at 94°C for 1 min, followed by 98°C for 5 s, 55°C for 5 s, and 72°C for 10 s for 30 cycles. The primers used in this study were as follows: IL-6, forward 5′- CATATAAAATAGTCCTTGCTACCCCAACT -3′ and reverse 5′- CCACTCCTTCTGTGACTCTAACTTGTC -3′; TNF-*α*, forward 5′- GGCAGGTCTACTTTGGAGTCATTG -3′ and reverse 5′- ACATTCCGGGATCCAGTGAGTTCCG -3′; and *β*-actin, forward 5′- TCAGCAAGCAGGAGTACGATGA -3′ and reverse 5′- TGCGCAAGTTAGGTTTTGTCAA -3′. The PCR products were separated by electrophoresis on a 2% agarose gel and stained with ethidium bromide.

### 2.9. Statistical Analyses

Data were presented as the mean ± standard deviation (SD) for three independent experiments using different batches of cells. The significant differences in the mean values were assessed using the unpaired Student's *t*-test. Significance was defined as *p* < 0.05 ( ^*∗*^) and *p* < 0.01 ( ^*∗∗*^) versus the appropriate control group.

## 3. Results

### 3.1. Inhibitory Effect of PSPLE on the Viability of 3T3-L1 Adipocytes

**Figure 1:**
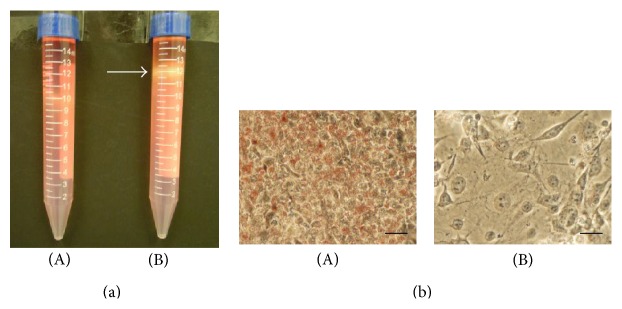
Separation of adipocytes and preadipocytes from differentiated 3T3-L1 cells by Percoll density gradient centrifugation. 3T3-L1 preadipocytes were grown to confluence and then in the absence (A) or presence (B) of adipogenic agents, which induced cellular differentiation. The differentiated 3T3-L1 cells, which included both adipocytes (arrow) and preadipocytes, were separated by Percoll gradient centrifugation (a). After centrifugation, adipocytes (A) and preadipocytes (B) were cultured on collagen-coated dishes overnight and then stained with Oil Red O (b). The scale bar equals 30 *μ*m.

3T3-L1 preadipocytes were differentiated into adipocytes through treatment with adipogenic agents (insulin, DMX, and IBMX). However, not all preadipocytes could be transformed to adipocytes. The differentiated 3T3-L1 cells could be divided into 3 layers after Percoll density gradient centrifugation, whereas the preadipocytes did not separate (Figure 1(a)). The results indicated that the cells were distributed in the middle layer and the lower layer. In addition, these cells were stained with Oil Red O, which indicates that the cells in the middle layer were rich in oil droplets but that the cells in the lower layer were not labeled (Figure 1(b)). Accordingly, the differentiated 3T3-L1 cells consisted of adipocytes and preadipocytes that were distributed in the middle layer and the lower layer after Percoll density gradient centrifugation.

**Figure 2:**
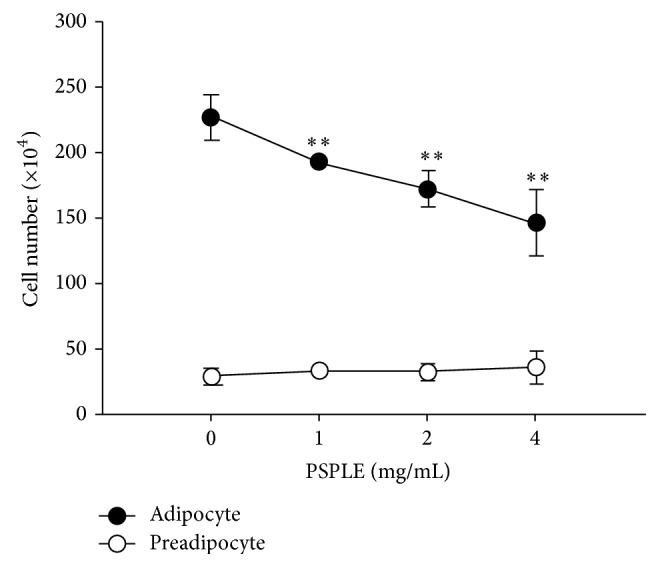
Effects of PSPLE on 3T3-L1 adipocyte and preadipocyte viability. The differentiated 3T3-L1 cells were treated with PSPLE (0, 1, 2, and 4 mg/mL) for 72 h, and both adipocytes and preadipocytes were separated by Percoll gradient centrifugation. The viability of the cells was determined by Trypan blue exclusion. The results are presented as the mean ± SD. ^*∗∗*^ *p* < 0.01, compared with 0 mg/mL PSPLE.

The differentiated 3T3-L1 cells were treated with several concentrations of PSPLE for 72 h and were then separated into adipocytes and preadipocytes using Percoll density gradient centrifugation. PSPLE reduced the number of adipocytes in a dose-dependent manner, as determined by Trypan blue exclusion. Compared to the control, the number of adipocytes was reduced by 17%, 25%, and 33% for the differentiated 3T3-L1 cells treated with 1, 2, and 4 mg/mL PSPLE, respectively. However, the number of preadipocytes was not decreased compared to the control in the differentiated cells cultured with various concentrations of PSPLE (Figure 2). Therefore, the results demonstrated that PSPLE possessed cytotoxic activity in adipocytes but not in preadipocytes.

### 3.2. PSPLE-Induced Apoptosis of Differentiated 3T3-L1 Cells

**Figure 3:**
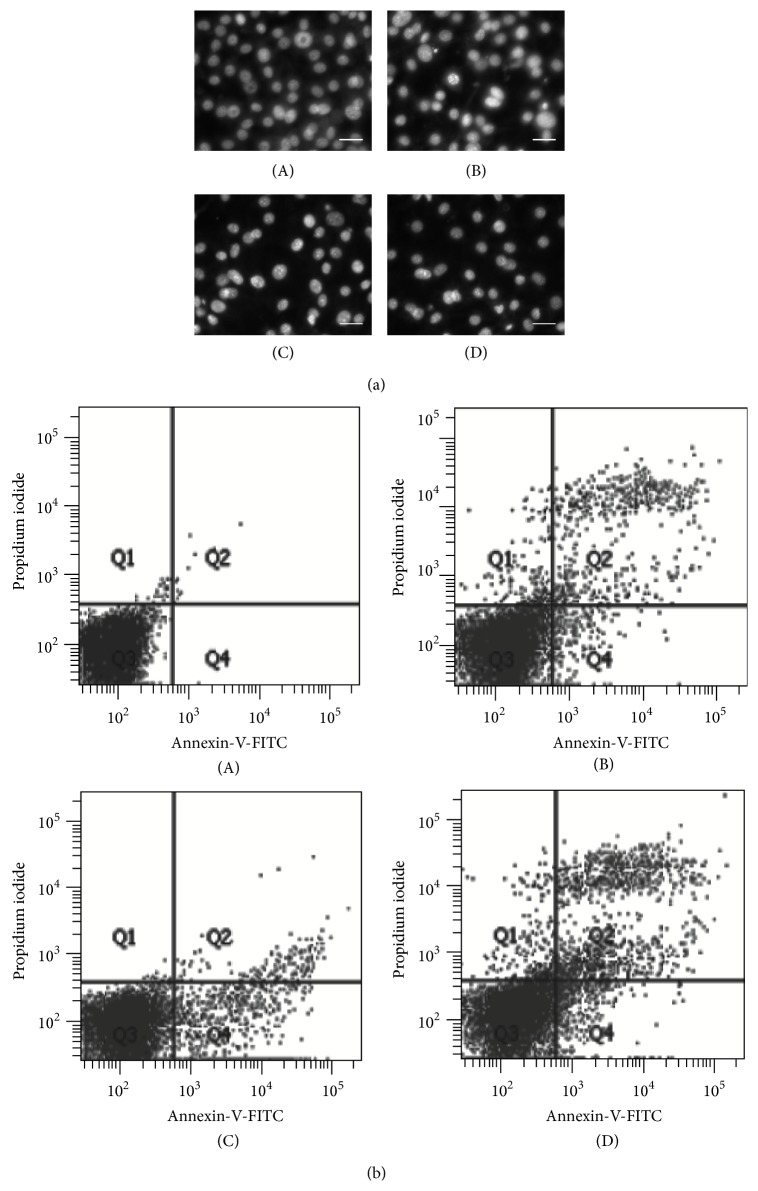
Determination of PSPLE-induced apoptosis of 3T3-L1 adipocytes. The differentiated 3T3-L1 cells were cultured with PSPLE at 0 (A), 1 (B), 2 (C), and 4 (D) mg/mL for 72 h. Then, the cells were stained with DAPI and were observed under a fluorescence microscope (a). In addition, the cells were incubated with FITC-conjugated annexin-V and propidium iodide (PI) and were measured by flow cytometry (b). Normal cells were annexin-V-negative and PI-negative (Q3); cells in early apoptosis were annexin-V-positive and PI-negative (Q4); cells in late apoptosis/necrosis were annexin-V-positive and PI-positive (Q2). The scale bar equals 30 *μ*m.

**Figure 4:**
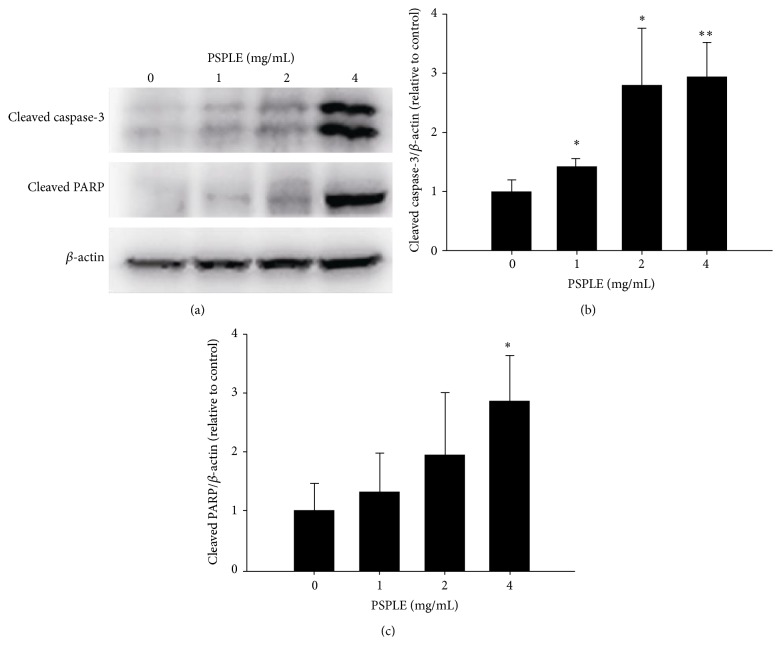
Effects of PSPLE on the expression of apoptosis-associated proteins in 3T3-L1 adipocytes. The differentiated 3T3-L1 cells were incubated with PSPLE (0, 1, 2, and 4 mg/mL) for 72 h. The cells were harvested and analyzed for cleaved caspase-3, cleaved PARP, and *β*-actin by western blot analysis (a). Quantitative densitometric analysis was used to calculate the values of cleaved caspase-3 (b) and cleaved PARP (c), which were normalized against *β*-actin. The results are presented as the mean ± SD. ^*∗*^ *p* < 0.05, ^*∗∗*^ *p* < 0.01, compared with 0 mg/mL PSPLE.

Our findings show that PSPLE had an inhibitory effect on adipocyte viability. We further investigated whether the reduction in cell number by PSPLE involved apoptosis, and the cells were stained using the fluorescent DNA-binding agents DAPI or PI, respectively. The results showed that cells treated with PSPLE demonstrated typical characteristics of apoptosis such as chromatin condensation, as determined by DAPI staining (Figure 3(a)). Additionally, PSPLE increased the number of cells in the sub-G1 phase of the cell cycle in a dose-dependent manner. The percentage of cells in the sub-G1 phase was 5.4%, 9.4%, and 14% at 1, 2, and 4 mg/mL of PSPLE-treatment, respectively (Table 1). Furthermore, annexin-V/PI double staining showed an increase in the number of cells in both early apoptosis and late apoptosis/necrosis compared to cells treated without PSPLE (Figure 3(b)). These results showed that the number of cells in early apoptosis was 3.2–10% for PSPLE treated cells and 2.1% for untreated cells. Furthermore, the number of cells in late apoptosis/necrosis was 5.2–9.5% for PSPLE treated cells and 1.5% for untreated cells (Table 2). We further explored the molecules associated with PSPLE-induced apoptosis. The results of immunoblot analysis revealed that PSPLE (4 mg/mL) significantly elevated the amount of the cleaved caspase-3 and PARP by 2.9-fold and 2.8-fold, respectively (Figure 4). These results suggest that PSPLE induced caspase-3-dependent apoptosis in 3T3-L1 adipocytes.

### 3.3. PSPLE-Induced Downregulation of Inflammation-Associated Genes in Differentiated 3T3-L1 Cells

**Figure 5:**
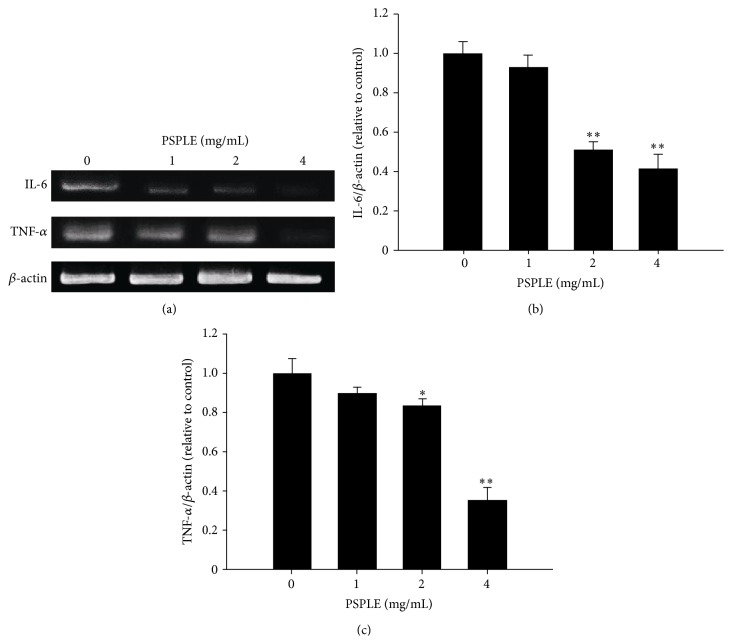
Effects of PSPL on the expression of inflammation-associated genes in 3T3-L1 adipocytes. The differentiated 3T3-L1 cells were exposed to PSPLE at concentrations of 0, 1, 2, and 4 mg/mL for 72 h. The expression levels of IL-1, TNF-*α*, and *β*-actin were measured by RT-PCR (a). Quantitative densitometric analysis was used to calculate the values of IL-1 (b) and TNF-*α* (c) that were normalized against *β*-actin. The results are presented as the mean ± SD. ^*∗*^ *p* < 0.05, ^*∗∗*^ *p* < 0.01, compared with 0 mg/mL PSPLE.

Adipocytes possess an endocrine role that modulates systemic metabolism [22]. In addition, adipocytes are the source of proinflammatory molecules, such as TNF-*α* and IL-6 [23]. Therefore, we investigated whether PSPLE modulated the expression of IL-6 and TNF-*α*. As illustrated in Figure 5, we found that TNF-*α* and IL-6 mRNA levels were reduced in a dose-dependent manner with significant reductions of 49% and 17% at 2 mg/mL and 59% and 65% at 4 mg/mL, respectively. These results indicated that PSPLE suppressed the expression of IL-6 and TNF-*α* in the differentiated 3T3-L1 cells.

## 4. Discussion

3T3-L1 cells have widely been used to study adipogenesis and the biochemistry of adipocytes [24]. Conversion of 3T3-L1 cells from preadipocytes to adipocytes occurs through treatment with adipogenic agents, including dexamethasone (DEX), isobutylmethylxanthine (IBMX), and insulin [25]. However, the differentiation efficiency was not consistent using different cell passages that had been stored in liquid nitrogen [26]. In addition, different brands of insulin could also affect the differentiation efficiency (data not shown). It is important that a suitable level of differentiation of preadipocytes to adipocytes is obtained for use in research. We evaluated the differentiation efficiency as determined by density gradient centrifugation, which can separate adipocytes and preadipocytes at different densities (Figure 1). Therefore, this research used differentiated 3T3-L1 cells at a differentiation level of approximately 90%. Additionally, the proportion of adipocytes was 90%, 86%, 84%, and 82% at 0, 1, 2, and 4 mg/mL PSPLE, respectively, when differentiated 3T3-L1 cells were treated for 72 h (Figure 2). PSPLE reduced the proportion of adipocytes rather than preadipocytes, suggesting a differential susceptibility to PSPLE-induced cytotoxicity of adipocytes and preadipocytes. Our findings were consistent with a previous study showing that PSPLE-treatment did not induce cytotoxicity but inhibited the proliferation of 3T3-L1 preadipocytes [18]. Additionally, reports indicate that preadipocytes acquire a relative resistance to apoptosis as they differentiate [8]. Herein, we speculate that PSPLE include specific ligands which play a central role in apoptosis. The specific ligands may induce upregulation of Fas result in differentiated 3T3-L1 cell-specific killing. However, the bioactive compound of PSPLE that induced differentiated 3T3-L1 cell apoptosis requires further identification.

Recently, a few groups reported that the induction of cell death in adipocytes through apoptosis may be a valid approach for the prevention and treatment of obesity [27]. However, Alkhouri et al. demonstrated that the inhibition of adipocyte apoptosis may be a strategy for the treatment of obesity-associated metabolic complications [28]. Sun and colleagues documented that determining whether adipocytes priorly undergo a simple necrotic death or apoptosis is almost impossible* in vivo* due to the technical difficulties of reproducibly demonstrating apoptosis in adipocytes [3]. Our data indicated that PSPLE induced adipocyte death through a caspase-3-dependent apoptotic pathway (Figure 4); however, necrosis of the cells cannot be excluded (Table 2). Accordingly, we speculate that PSPLE could be a potent protector against hyperplasia, which results from induced adipocyte death and inhibitory proliferation of preadipocytes.

Adipose tissue is an important endocrine organ that secretes a variety of adipokines implicated in the regulation of energy metabolism, insulin resistance, and metabolic syndrome. Serum adipokine levels increase in proportion to adiposity. Reduction of adipose tissue mass correlates with decrease in serum adipokine levels [29]. Proinflammatory cytokines, such as IL-6 and TNF-*α*, are adipokines that have been associated with the development of insulin resistance and type 2 diabetes [30, 31]. Our data indicate that PSPLE-treated 3T3-L1 adipocytes downregulated the expression of IL-6 and TNF-*α* (Figure 5). However, 3T3-L1 adipocytes are a model for the study of adipocytes; they do not represent the complexity of adipose tissue. Therefore, further* in vivo* studies of the efficacy of PSPLE regulated inflammation-associated genes are necessary. In addition, other studies have demonstrated that consuming a high-PSPL diet can decrease exercise-induced plasma IL-6 concentrations and oxidative damage in healthy adults [13]. Additionally, PSPL are rich in flavonoids with antioxidant properties, which have known anti-inflammatory effects [12, 14]. We have detected that PSPLE possessed free radical scavenging activity that was evaluated by using 1,1-diphenyl-2-picrylhydrazyl (DPPH) assay (data not shown). Therefore, we speculate that the antioxidant activities of PSPLE may suppress the expression of proinflammatory cytokines. Additionally, the bioactive compound of PSPLE that regulate proinflammatory cytokines requires further identification.

## 5. Conclusions

Our data demonstrated that PSPLE possessed cytotoxicity in 3T3-L1 adipocytes but not in preadipocytes. PSPLE induced caspase-3-dependent apoptosis in differentiated 3T3-L1. In addition, PSPLE treatment caused the downregulation of IL-6 and TNF-*α* expression. These results indicated that PSPLE could modulate adipose tissue mass and inflammation. However, further* in vivo* investigations into the efficacy of the antiobesity and anti-inflammatory effects of PSPLE are necessary.

## Figures and Tables

**Table 1:** Effect of PSPLE on the cell cycle distribution of differentiated 3T3-L1 cells.

PSPLE	Sub-G1 phase	G0/G1 phase	S phase	G2/M phase
(mg/mL)				
0	0%	83 ± 1.8%	9.1 ± 2.6%	7.9 ± 1.3%
1	5.4 ± 2.4%^*∗*^	80 ± 0.8%^*∗*^	6.6 ± 0.7%	7.8 ± 2.2%
2	9.4 ± 0.9%^*∗∗*^	81 ± 1.6%	3.5 ± 0.9%^*∗*^	6.8 ± 2.0%
4	13 ± 1.4%^*∗∗*^	80 ± 1.8%	2.4 ± 0.8%^*∗*^	5.7 ± 0.5%

Differentiated 3T3-L1 cells were incubated with the indicated concentrations of PSPLE for 72 h, stained with PI, and then measured by flow cytometry. The results are presented as the mean ± SD. ^*∗*^ *p* < 0.05, ^*∗∗*^ *p* < 0.01, compared with 0 mg/mL PSPLE.

**Table 2:** Quantification of apoptosis in differentiated 3T3-L1 cells exposed to PSPLE.

PSPLE	Normal cells	Cells in the early apoptosis	Cells in the late apoptosis/necrosis
(mg/mL)			
0	96 ± 2.6%	2.1 ± 1.8%	1.5 ± 1.0%
1	89 ± 2.6%^*∗∗*^	3.2 ± 0.5%	5.2 ± 0.8%^*∗∗*^
2	83 ± 6.0%^*∗∗*^	10 ± 1.7%^*∗∗*^	6.6 ± 4.2%
4	78 ± 3.3%^*∗∗*^	10 ± 3.4%^*∗∗*^	9.5 ± 1.9%^*∗∗*^

Differentiated 3T3-L1 cells were treated with the indicated concentrations of PSPLE for 72 h. Cells were harvested and incubated with FITC-conjugated annexin-V and PI and were then measured by flow cytometry. The results show the percentage of cells in various stages, and the values are presented as the mean ± SD. ^*∗∗*^ *p* < 0.01, compared with 0 mg/mL PSPLE.
